# Liposarcoma Arising in the Foot: A Case Report

**DOI:** 10.1155/2009/630203

**Published:** 2009-11-08

**Authors:** Toshihiro Matsuo, Takashi Sugita, Shoji Shimose, Tadahiko Kubo, Yuji Yasunaga, Mitsuo Ochi

**Affiliations:** ^1^Department of Artificial Joints and Biomaterials, Graduate School of Biomedical Sciences, Hiroshima University, 1-2-3 Kasumi, Minami-ku, Hiroshima 734-8551, Japan; ^2^Department of Orthopaedic Surgery, Hiroshima Prefectural Hospital, 1-5-54 Ujinakanda, Minami-ku, Hiroshima 734-8530, Japan; ^3^Department of Orthopaedic Surgery, Graduate School of Biomedical Sciences, Hiroshima University, 1-2-3 Kasumi, Minami-ku, Hiroshima 734-8551, Japan

## Abstract

Liposarcoma is categorized as a soft tissue sarcoma and most commonly appears in the lower extremities and rarely in the foot during adulthood. We present a very rare case report of a primary well-differentiated liposarcoma arising in the foot on a 60-year-old female. Marginal resection of the tumor with metatarsal ray amputation was eventually performed. The patient's postoperative course was uneventful without recurrence 5 years after the original operation. The authors review the literature and also report on the low incidence of this tumor arising in the foot.

## 1. Case Presentation

**Figure 1:**
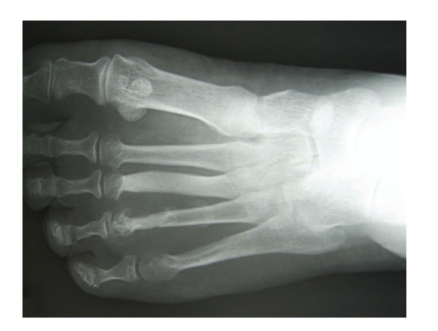
Plain radiograph at admission.

**Figure 2:**
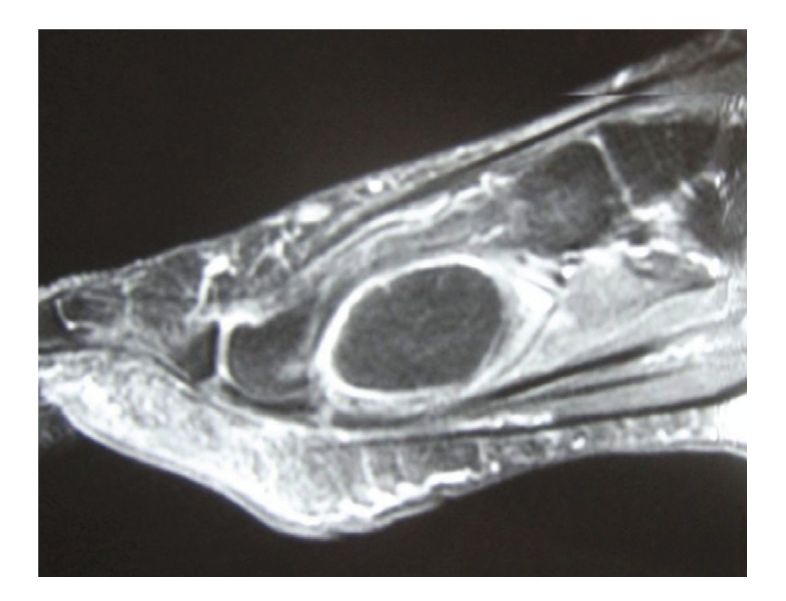
Sagittal gadolinium-enhanced MRI at admission.

**Figure 3:**
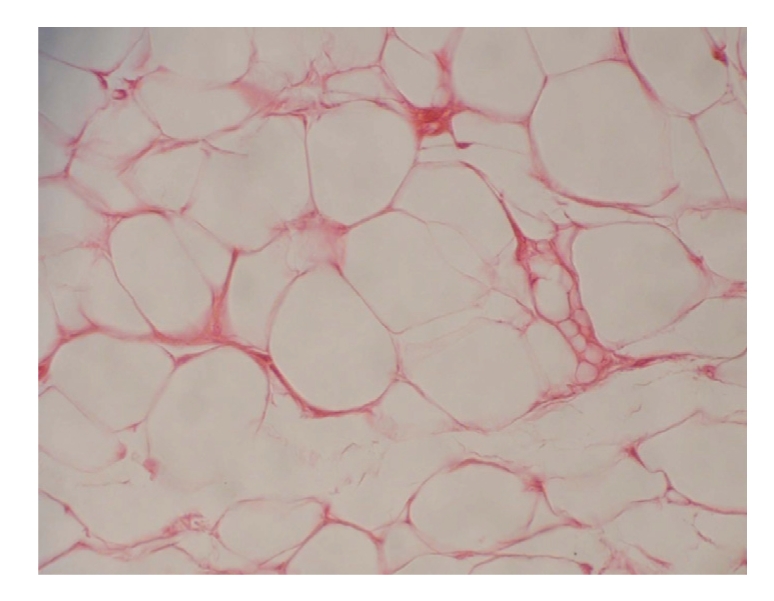
Photomicrograph. Well-differentiated liposarcomas composed of fat cells of a variety of sizes and shapes. H and E stain (×400).

A 60-year-old female presented to our institution with a history of a three year slowly growing mass to the dorsal aspect of the left foot. Prior to her visit, the patient had an excision of the soft tissue mass in a different facility but without any histopathological analysis. Patient did have a recurrence of the soft tissue mass and eventually presented to our institution four years after the original surgery. Plain foot radiographs were obtained and revealed cortical irregularities of the third and fifth metatarsals suggesting possible occurrence of the local soft tissue invasion (Figure 1). Magnetic resonance imaging demonstrated the existence of a soft tissue mass arising in the dorsal-to-plantar side of the left foot and partially invading into the bone cortex. On T1 and T2 weighted images, the signal intensities of the soft tissue mass were found to be significantly high and, after administration of a contrast medium, peripheral enhancement was observed (Figure 2). After an open biopsy, the mass was diagnosed as a well-differentiated liposarcoma (Figure 3). Marginal resection of the tumor along with a ray amputation including the fourth metatarsal and the cortices of the third and the fifth metatarsal bones, was performed. At five years postoperatively, the patient is able to ambulate without any assistance or discomfort. Her ISOLS scale was estimated to be 100% in accordance with the Enneking's criteria [1] and no local recurrence has been detected at her last visit.

## 2. Discussion

Liposarcoma is considered as a secondary common sarcoma frequently accompanied by malignant fibrous histiocytoma. Liposarcomas are well known to arise in the lower extremities, particularly in the thigh [2, 3], but rarely in the foot. Among the 1067 liposarcoma cases analyzed by Armed Forces Institute of Pathology (AFIP), there were no patients with liposarcoma located in the foot [4]. In a review of the English literature, only few cases of liposarcomas in the feet have been reported [5–7].

There are considerable histologic and imaging similarities between lipomas and a well-differentiated liposarcomas. A well-differentiated liposarcoma closely simulates the lipoma presentation except for a scattering of lipoblasts with irregulary shaped hyperchromatic nuclei and one or more lipid droplets in the cytoplasm [4]. In the previous reports, features that suggest malignancy included presence of thick septa, presence of nodular and/or globular or nonadipose mass-like areas and decreased percentage of fat composition [8]. However, our case did not indicate these findings and therefore it was difficult to distinguish from lipoma by the medical imaging although scalloping of the outer cortex of the bone was seen on plain radiographs.

A type of dedifferentiated liposarcomas has been well documented to develop as a late complication of a pre-existing well-differentiated liposarcoma after a long interval. In addition, recurrent well-differentiated liposarcomas showed a tendency of exhibiting several characteristics closely associated with dedifferentiation [9, 10]. Such dedifferentiated liposarcomas were reported to behave as a high-grade sarcoma: a local recurrence rate, 41%; a metastatic rate, 17%; a disease-related mortality, 28% [10].

## 3. Conclusion

Foot liposarcoma presents with a very low incidence and a high index of suspicion is required for its early diagnosis and prevention of its clinical entity and grade with proper surgical resection and treatment.
